# Undergraduate nursing students’ knowledge of and attitudes toward people with alzheimer’s disease

**DOI:** 10.1186/s12877-022-03389-6

**Published:** 2022-08-22

**Authors:** Ma’en Aljezawi, Mohammad Al Qadire, Mohammad Suliman, Omar Al Omari, Atika Khalaf

**Affiliations:** 1 College of Nursing, Sultan Qaboos University, P.O. Box 66, PC 123 Muscat, Sultanate of Oman; 2 Faculty of Nursing, Al Al-Bayt University, P.O. Box 130040, Mafraq, 25113 Jordan; 3 Faculty of Health Sciences, Kristianstad University, Elmetorpsvägen 15, 291 88 Kristianstad, SE Sweden

**Keywords:** Alzheimer’s disease, Knowledge, Student, Nursing, Attitudes

## Abstract

As the population ages, the number of people living with Alzheimer's disease is expected to grow; consequently, nursing students are expected to care for more people with Alzheimer's disease in their future careers. Exploring nursing students' level of knowledge and attitudes is essential here to fill any knowledge gap and enhance attitudes. For this reason, the current study aimed to measure the knowledge of and attitudes toward people living with Alzheimer's disease among undergraduate Jordanian nursing students. A descriptive cross-sectional design was utilized. Data were collected through an online questionnaire consisting of the Alzheimer's Disease Knowledge Scale (ADKS) and Dementia Attitudes Scale (DAS). A third part contained questions about previous formal education about Alzheimer's disease, reading Alzheimer's research, and the need for formal education about Alzheimer's disease. The study targeted all undergraduate Jordanian nursing students. A total of 275 students agreed to participate and completed the questionnaire. Jordanian nursing students had low knowledge regarding people living with Alzheimer's disease, with a mean ADKS score of 18.3 out of 30; however, their attitudes were positive, with a mean DAS score of 91 out of 140. There was no statistical difference in attitude or knowledge between different academic levels. The majority of students (90.5%) expressed their desire to have a formal education regarding Alzheimer's disease. Knowledge regarding people with Alzheimer's disease could be improved through training and education. Positive attitudes reported by students could augment the learning process.

## Introduction

Alzheimer's disease (AD) is a slowly progressive neurological disease that causes significant declines in cognitive abilities and motor function [1]. Alzheimer’s disease constitutes the majority of illnesses under the giant umbrella of dementia, accounting for 60% to 80% of its causes [2]. .People with AD experience many symptoms that worsen as time passes, such as difficulty remembering, apathy, depression, impaired communication, difficulty speaking, confusion, and poor judgment [3]. These symptoms usually occur before brain pathology, in which amyloid plaques and tau tangles cause death to specific neurons [4]. AD develops as an interaction of complex risk factors; some are non-modifiable, such as age, family history, genetic mutations, and others are modifiable [5]. The Lancet Commission reported 12 modifiable risk factors, namely: less education, hypertension, air pollution, hearing impairment, smoking, obesity, depression, excessive alcohol consumption, physical inactivity, diabetes, traumatic brain injury, and low social contact [6].

It can be noticed from the global figures that 5% to 8% of people above 60 years living with dementia, with an estimated 50 million persons living with dementia [7]. This number is expected to increase by 10 million annually to reach 82 million by 2030 and 150 million by 2050, eventually, this will lead to an increase in healthcare costs [1, 8]. To compensate for this increase in prevalence and cost, nursing students and nurses require dementia knowledge and competencies to promote a positive health outcome [9]. Having said that, people with dementia can also experience joy, humor, and positive aspects of life [10]. In Jordan, where the current study took place, there are no reported epidemiological figures about AD; however, the number of older people is expected to increase, thus increasing the chance for more AD cases to develop [11].

As can be noticed from the figures mentioned above, there is an upward shift in the number of people living with AD globally and in Jordan [12]. Student nurses will come in direct contact with these individuals in their future careers, and they may not have the required knowledge to provide care for such people [13]. In this domain, a study conducted in Spain found moderate knowledge of AD among nursing students. Seniority in the nursing program, exposure to patients with AD, and formal education about AD received during enrollment in the program were factors that are associated with better AD knowledge [14]. However, this is not always the case. A different study conducted on undergraduate nursing students found insufficient AD knowledge, also the level of knowledge and age of participants correlated negatively [15]. The research reports mentioned above display contradictory findings, which could result from different inclusion criteria and sampling methods. The first study used a convenience sampling method and included all students, while the second study used randomization and included only third- and fourth-year students. A different cross-sectional survey with a sample that included all students from different levels found a satisfactory AD knowledge level, which was positively associated with students' age [16]. One explanation for these results could be related to a dedicated dementia course that took place later in the curriculum (e.g., in the third or fourth year), which provided the senior students with formal learning opportunities.

Students who reported poor knowledge will probably face additional challenges during their future careers [17]. Consequently, nursing schools will have to adapt their curricula and add specialized courses and training to enhance the best AD care [18].

Knowledge is not the only requirement needed to provide the best care for persons living with A.D.; nursing students need positive attitudes as well [19]. For this reason, several studies have assessed nursing students’ attitudes toward people with AD and their knowledge of the disease, with different results. For example, a systematic review reported positive attitudes among nursing students from different levels [9]. In contrast, a different longitudinal study reported negative to neutral attitudes in the first- and second-year nursing students; the attitudes changed to moderately positive when the students were in their third and fourth years. A negative attitude could result from exposure to negative role modeling during the students' clinical training [20]. It might also be the case that nursing staff in the clinical area are unable to provide adequate mentoring to students as they lack AD knowledge themselves [21]. Therefore, it is essential to provide adequate knowledge and guidance for nursing students in order to shift their attitudes toward the positive side [18].

In summary, the number of people living with AD is expected to rise globally, and, in Jordan, this requires a qualified nursing workforce to provide the best care for them [12]. It is noted in the literature that there is a knowledge gap regarding AD among nursing students and a noticeable negative attitude toward people living with AD [22]. Apart from academic curriculums, nursing students need to internalize a positive attitude, also they must have dementia competencies to provide patient-centered care and respond appropriately to behavioral and psychological symptoms [9]. A plethora of international studies exists that explore student nurses’ knowledge and attitude toward people living with AD. However, to the best of our knowledge, there are no published studies in Jordan that explored the same area. Consequently, this study aimed to investigate knowledge of and attitude toward people living with AD among Jordanian nursing students and explore factors affecting these concepts. Accordingly, two research questions were formulated. First, ‘What is the level of knowledge and attitudes among Jordanian nursing students toward people living with AD?’ Second, ‘Does the academic level (senior in student tenure) of nursing students affect their knowledge and attitude toward people living with Alzheimer's disease?’

## Methods

### Design and setting

The study utilized a descriptive cross-sectional design. This study is about exploring AD knowledge and attitudes among Jordanian university nursing students (Baccalaureate degree) and the effect of the academic level (represented by years from the first-year level up to the fourth-year level) on their knowledge and attitude. As the study aimed to explore Jordanian student nurses’ knowledge and attitude toward AD, it targeted all Jordanian student nurses from all Jordanian universities. There are 16 nursing schools in Jordan, geographically scattered, all offering a four-year nursing degree (Baccalaureate degree). The curricula of the different nursing schools are approximately similar because they are based on “learning objectives and competencies” set by the Jordanian Council of Higher Education.

### Participants and procedure

The current study targeted all enrolled students in the participating nursing schools (4000 students approximately enrolled). Data collection was conducted online using the questionnaire mentioned in the next sub-section. Invitations along with an information sheet about the study and an electronic link to the questionnaire were posted on different participating nursing schools' formal Facebook pages, as each nursing school in Jordan has an official Facebook page used to communicate with students. The majority of students in each nursing school follow these pages to stay updated with any new information, as it is the only fast and credible method of communication with their faculty. To obtain sufficient responses, the questionnaire was re-posted weekly for a period of two months from June 2019 to July 2019. At the end of the survey period, all responses were exported to a statistical program, i.e., SPSS, and re-checked manually for any export errors that might have occurred. To prevent data duplication (the same student gives more than one answer), data entries with the same electronic ID were deleted. The required sample size was calculated using G* Power version 3.1., with an effect size of 0.25, and an Alpha error of 0.05, to determine the smallest sample size appropriate to detect a given test's effect at the desired level of significance. The power analysis gave a minimum sample size of 270 for an ANOVA test. When data were collected, a sample of 275 was obtained [23].

### Theoretical framework and Instruments

To achieve the aim of the study and explore relevant data, the ‘Promoting Excellence’ framework was used as a theoretical framework to guide the study. This framework outlines the knowledge and skills required from a health care professional to provide specific intervention and care for people with dementia [24]. The framework is built based on the lowest level of knowledge that must be possed to provide competent care for people living with dementia and their families [25]. The framework focuses on areas to be improved on personal and organizational levels. On a personal level, it can help the nursing students to realize gaps in their knowledge through questions in the survey so they can fill these gaps. As a result, they will feel more confident in themselves and their skills, which will eventually be reflected as a more positive attitude toward their future patients with AD. On the organizational level, the educational sector can benefit from the gaps found in the students’ knowledge in order to inform the content of the education and training they provide and shape the design and delivery of future focused vocational and professional education and training [26].

Therefore, the author designed a questionnaire (outlined below) that covers points outlined in the aforementioned framework and helps in achieving outcomes of care. In order for the questionnaire to be valid and clear, the primary investigator prepared a draft of the questionnaire based on literature that was distributed to members of an advisory group (three dementia clinical nurse specialists and two university professors who are experts in dementia) to ensure that the survey included all key questions identified in the study aims. The face validity of the survey was tested by distributing it to members of the advisory group. Feedback regarding the questionnaire was used to improve clarity. The reliability analysis yielded Cronbach Alpha values of 0.79 for the knowledge and 0.76 for the attitude scales in this study. Additional feedback about the time needed to complete the questionnaire and clarity, a link to the online questionnaire was sent to ten nursing students from different levels. The average time needed for them to complete the questionnaire was ten minutes. Their feedback for clarity was also considered to prepare the final version of the questionnaire, which consisted of three parts:

1. Questions about demographical data, including the academic level for the participants represented by years from first to fourth year (senior in student tenure), age, gender, in addition to four questions. The first asks whether the participant has previously had any formal education about A.D., the second asks whether they have read any research about AD and from how long, and the third ask about the period since the participant read research about AD, the fourth asks if they think they need any formal training regarding AD (see Table 1 for full details).
2. Alzheimer's Disease Knowledge Scale (ADKS): The scale consists of 30 true/false questions that cover seven areas of knowledge regarding A.D.; these are: assessment (4 questions), symptoms (4 questions), risk factors (6 questions), impact on life (4 questions), care, management (4 questions), and treatment (4 questions) (Table 2). The scale has a score ranging from 0 to 30, and it is suitable to use with students and healthcare workers and takes usually 5-10 minutes to complete [27]. In the current study, the survey was distributed online and the mean time to complete it was 10 minutes calculated based on the electronical measurements. The scale has good test-retest reliability (median *r* =.78), internal consistency reliability (alpha = .80), and validity (median *r* = .71) [28].
3. The Dementia Attitudes Scale (DAS): This is a 20-item scale, with 7-point Likert scale responses, ranging from strongly agree to strongly disagree. It is designed to reveal attitudes toward AD and related dementia. The Likert-scale answers give a total score ranging from 20 to 140. Items in the DAS represent two major domains, namely: “dementia knowledge” and “social comfort”, with a score ranging from 10 to 70 for each one [29]. The scale has a Cronbach’s alpha coefficient ranging from 0.83-0.85, which represents strong reliability. It also has good validity compared with other scales that measure attitude, making it a suitable research tool [30]. To calculate the total DAS score, the negatively worded items (items: 2, 6, 8, 9, 16, 17) were reversely coded. The two previously mentioned scales were used in their original form, as the language of teaching and instruction in Jordanian nursing schools is English.

**Table 1:** Students’ characteristics (*N* = 275)

	n (%)
**Age (mean±SD)**	21.1±2.9
**Gender**	
Male	76 (27.6)
Female	199 (72.4)
**Academic level**	
First year	42 (15.3)
Second year	44 (16.0)
Third year	72(26.2)
Fourth year	117 (42.5)
**Previous training in AD care**	
Yes	74 (26.9)
No	201 (73.1)
**Previous education about AD care**	
Lecture	143 (52.0)
University course	33 (12.0)
Conference	2 (0.7)
Workshop	6 (2.2)
Never	91 (33.1)
**Period since you read research about AD**	
< one month	19 (6.9)
1-3 months	26 (9.5)
4-6 months	29 (10.5)
> 6 months	77 (28.0)
Never	124 (45.1)
**Do you need education about AD**	
Yes	294 (90.5)
No	26 (9.5)

**Table 2:** Distribution of students’ answers for the ADKS (*N* = 275)

Question (answer)	Correct answer	%
**Life impact**		
1-People with AD are particularly prone to depression (T)	237	86.2
2-Most people with AD live in nursing homes (F)	139	50.5
3-It is safe for people with AD to drive, as long as they have a companion in the car at all times (F)	180	65.5
**Risk factors**		
4-It has been scientifically proven that mental exercise can prevent a person from getting AD (F)	31	11.3
5-People in their 30s can have AD (T)	160	58.2
6-Having high cholesterol may increase a person’s risk of developing AD (T)	127	46.2
7-Prescription drugs that prevent AD are available (F)	196	71.3
8-Having high blood pressure may increase a person’s risk of developing AD (T)	148	53.8
9-Genes can only partially account for the development of AD (T)	227	82.5
**Symptoms**		
10-Tremor or shaking of the hands or arms is a common symptom in people with AD (F)	166	60.4
11-Trouble handling money or paying bills is a common early symptom of AD (T)	85	30.9
12-One symptom that can occur with AD believes that other people are stealing one ’s things (T)	156	56.7
13-Most people with AD remember recent events better than things that happened in the past. (F)	172	62.5
**Treatment and management**		
14-People whose AD is not yet severe can benefit from psychotherapy for depression and anxiety. (T)	205	74.5
15-Poor nutrition can make the symptoms of AD worse. (T)	224	81.5
16When a person has AD, using reminder notes is a crutch that can contribute to decline. (F)	199	72.4
17-AD cannot be cured. (T)	158	57.5
**Assessment and diagnosis**		
18-When a person with AD becomes agitated, a medical examination might reveal other health problems that caused the agitation. (T)	211	76.7
19-If trouble with memory and confused thinking appears suddenly, it is likely due to AD. (F)	199	72.4
20-Symptoms of severe depression can be mistaken for symptoms of AD. (T)	139	50.5
21-AD is one type of dementia. (T)	232	84.4
**Caregiving**		
22-People with AD do best with simple, instructions given one step at a time. (T)	176	64.0
23-When people with AD begin to have difficulty taking care of them, caregivers should take over right away.(F)	16	5.8
24-If a person with AD becomes alert and agitated at night, a good strategy is to try to make sure that the person gets plenty of physical activity during the day. (T)	176	64.0
25-When people with AD repeat the same question or story several times, it is helpful to remind them that they are repeating themselves. (F)	123	44.7
26-Once people have AD, they are no longer capable of making informed decisions about their own care. (F)	149	54.2
**Course of the disease**		
27-After symptoms of AD appear, the average life expectancy is 6 to 12 years. (T)	180	65.5
28-In rare cases, people have recovered from AD. (F)	142	51.6
29-A person with AD becomes increasingly likely to fall down as the disease gets worse. (T)	229	83.3
30-Eventually, a person with AD will need 24-hour supervision. (T)	257	93.5

*AD* Alzheimer’s disease, *T* True, *F* False

### Statistical analysis

Data analysis took place using IBM SPSS Statistics version 20.0. Frequencies, mean (M), and standard deviation (S.D.) were used to describe the sample variables. One-way Analysis of Variance (ANOVA) with a post-hoc test (Tukey test) was used to explore the different ADKS scores and DAS between different academic levels. If the difference was significant, a post-hoc technique was used. Pearson correlation was used to determine if there is a correlation between mean ADKS scores and mean DAS scores. The significance level was set to 0.05.

### Ethical considerations

The pinicipal investigator's home university and the participating nursing schools granted the ethical approval to conduct the current study. The online survey did not ask for anything that could lead to the participants being identified, therefore, the individual information of the participants is not collected and they were anonymous. Students were instructed that participation in the study was entirely voluntary. Filling in the online questionnaire was proof of consent.

## Results

### Characteristics of the sample

As shown in Table 1, the total number of nursing students' responses was 275, with a mean age of 21.1 years (*SD*= 2.9). The majority of respondents were females (72.4%). The largest proportion of participants was from the fourth year (42.5%); the lowest was from the first year (15.3%). Only 12% of the students were enrolled in a course covering subjects related to dementia, and 45% had not read any research about dementia. Additionally, 90.5% of the participating students responded with “yes” when asked if they need formal education about A.D. No data was partially missing, as the questionnaire was electronic and did not allow for any incomplete submissions.

### ADKS scores

Table 2 shows the frequency and percentage of the students' correct answers to the ADKS questions. Responses were grouped according to the seven areas of the ADKS described previously under the methods section. The question that was most often answered correctly (93.5%) was question number 30, “Eventually, a person with AD will need 24-hour supervision”. The question that was least answered correctly (5.8%) was question number 23, “When people with AD begin to have difficulty taking care of them, caregivers should take over right away”.

Table 3 shows ADKS mean scores for the total sample and the four academic levels. The total sample's overall ADKS score was 18.3 out of 30 (equal to 61%). Using one-way ANOVA (Table 3), the difference in the total ADKS score between the four academic levels was not statistically significant (*p* > 0.05). Furthermore, there was no statistical difference in ADKS sub-scores between the four academic levels (*p* > 0.05), except for the “course of the disease”, which was significant (*p* = 0.002, *F*=5). Post-hoc comparison using the Tukey test indicated that fourth-year students had a significantly higher score for the “course of the disease” than other academic levels. However, the effect size for this difference was small (*Eta squared*=0.04).

**Table 3:** ADKS mean scores

	Sample M (SD)	1^**st**^year (***n*** = 42) M (SD)	2^**nd**^year (***n*** = 44) M (SD)	3^**rd**^year (***n*** = 72) M (SD)	4^**th**^year (***n*** = 117) M (SD)	F	P	Post-hoc difference
**Life impact (score out of 3)**	2 (0.7)	2.1 (0.7)	1.8 (0.7)	2 (0.7)	2 (0.7)	0.7	0.5	No difference
**Risk factors (score out of 6)**	3.2 (1.1)	3.5 (1.1)	3.2 (1)	3.3 (1.1)	3 (1.2)	2.6	0.06	No difference
**Symptoms (score out of 4)**	2.1 (0.8)	2.1 (0.7)	2.2 (0.8)	2.1 (1)	2 (0.9)	0.5	0.5	No difference
**Treatment and management (score out of 4)**	2.8 (0.8)	2.8 (0.8)	2.7 (0.9)	2.8 (0.8)	2.9 (0.9)	0.6	0.5	No difference
**Assessment and diagnosis (score out of 4)**	2.8 (0.8)	3 (0.8)	2.8 (0.7)	2.6 (0.8)	2.8 (0.8)	1.7	0.1	No difference
**Caregiving (score out of 5)**	2.3 (1)	2.5 (1)	2.2 (0.8)	2.4 (1)	2.2 (1)	1.8	0.1	No difference
**Course of the disease (score out of 4)**	2.9 (0.9)	2.6 (0.8)	2.5 (0.8)	3 (1)	3.1 (0.8)	5	0.002^a^	4>1,2,3
Total ADKS (score out of 30)	18.3 (2.7)	18.9 (2.7)	17.7 (2.6)	18 (2.8)	18.1 (2.7)	1.6	0.1	No difference

^a^significant

### DAS scores

Concerning student nurses' attitudes toward people with A.D., Table 4 shows these attitudes in the form of a mean DAS score. The overall mean DAS score for the sample was 91 out of 140 (equivalent to 65%). The mean score for the comfort sub-domain was 35.2 out of 70 (equal to 50%). The knowledge sub-domain had a score of 48.8 out of 70 (equivalent to 69%). The mean total DAS score for the four academic levels was close, ranging from 89.9 to 93.8. One-way ANOVA revealed that the difference in the total DAS scores and the difference in the comfort and knowledge scores was not statistically significant (*p* > 0.05). Results from Pearson correlation indicated that the mean ADKS score and the mean DAS score were found to be positively correlated r(275) = .19, *p* < .01.

**Table 4:** Students’ attitude towards dementia (DAS scores)

Item	Sample M^**a**^ (SD^**b**^)	1^**st**^year (***n*** = 42) M (SD)	2^**nd**^year (***n*** = 44) M (SD)	3^**rd**^year (***n*** = 72) M (SD)	4^**th**^year (***n*** = 117) M (SD)
***1*-**It is rewarding to work with people who have ADRD^c^.	4.3 (1.6)	4.5 (1.6)	4.3 (1.5)	4.2 (1.4)	4.3 (1.7)
***2***-I am afraid of people with ADRD^f^.	5.8 (1.4)	6.2 (1.2)	5.6 (1.6)	5.9 (1.3)	5.7 (1.4)
***3-***People with ADRD can be creative.	4.4 (1.7)	4.4 (2)	4.7 (1.6)	4.3 (1.6)	4.4 (1.8)
***4*-**I feel confident around people with ADRD.	3.4 (1.6)	3.9 (1.9)	3.3 (1.6)	3.4 (1.5)	3.3 (1.5)
***5*-**I am comfortable touching people with ADRD.	3.5 (1.6)	3.6 (1.8)	3.5 (1.4)	3.8 (1.7)	3.4 (1.6)
*6*-I feel uncomfortable being around people with ADRD^f^.	5.1 (1.6)	6 (1.2)	5.4 (1.6)	5 (1.7)	4.8 (1.6)
***7*-**Every person with ADRD has different needs.	5.1 (1.7)	4.9 (1.9)	5(1.7)	5.2 (1.6)	5.1 (1.7)
***8***-I am not very familiar with ADRD^f^.	3.5 (1.9)	3.1 (2)	2.8 (1.7)	3.4 (1.6)	3.9 (1.9)
***9***-I would avoid an agitated person with ADRD^f^.	4.1 (1.8)	4.5 (2.1)	4.2 (1.7)	3.9 (1.9)	4.2 (1.8 )
***10***-People with ADRD like having familiar things nearby.	5.1 (1.7)	5.1 (1.9)	4.9 (2)	5.3 (1.6)	5.1 (1.6)
***11***-It is important to know the past history of people with ADRD.	5.6 (1.7)	5.6 (1.8)	5.4 (1.8)	5.9 (1.5)	5.5 (1.8)
***12***-It is possible to enjoy interacting with people with ADRD.	4.4 (1.7)	4.7 (1.9)	4.4 (1.6)	4.5 (1.6)	4.3 (1.8)
***13***-I feel relaxed around people with ADRD.	3.3 (1.5)	3.3 (1.6)	3.5 (1.7)	3.1 (1.4)	3.5 (1.6)
***14***-People with ADRD can enjoy life.	4.2 (1.9)	4.1 (2.1)	4.5 (2.0)	4.3 (1.8)	4.1 (1.8)
***15***-People with ADRD can feel when others are kind to them.	5.2 (1.7)	5.2 (1.9)	5.1 (1.9)	5.5 (1.6)	5.1 (1.7)
***16***-I feel frustrated because I do not know how to help people with ADRD^f^.	3.8 (1.8)	3.4 (2)	3.8 (1.6)	4 (1.7)	3.9 (1.8)
***17***-I cannot imagine caring for someone with ADRD^f^.	4.8 (1.7)	5.6 (1.5)	4.8 (1.7)	4.6 (1.7)	4.7 (1.8)
***18***-I admire the coping skills of people with ADRD.	4 (1.7)	4.3 (1.8)	4.2 (1.6)	4 (1.8)	3.8 (1.6)
***19***-We can do a lot now to improve the lives of people with ADRD.	5.4 (1.8)	5.7 (1.8)	5.1 (1.8)	5.6 (1.6)	5.3 (1.8)
***20***-Difficult behaviors may be a form of communication for people with ADRD.	4.9 (1.8)	5 (1.8)	4.7 (1.8)	5 (1.7)	4.9 (1.8)
Total attitude scale score (out of 140)	91 (15.6)	93.8 (16.5)	89.9 (16.5)	91.6 (15.2)	90.1 (15.1)
Comfort score^d^ (out of 70)	35.2 (8.5)	34.4 (9.8)	36 (8.6)	35.6 (7.6)	34.9 (8.6)
Knowledge score^e^ (out of 70)	48.8 (13.4)	49.5 (15.8)	48 (13.5)	49.9 (12.7)	48 (13.2)

^a^Mean

^b^Standard deviation

^c^Alzheimer’s Disease and Related Dementia

^d^Items (1,2,4,5,6,8,9,13,16,17)

^e^Items (3,7,10,11,12,14,15,18,19,20)

^f^negatively worded items

## Discussion

This study aimed to measure undergraduate Jordanian nursing students' knowledge and attitudes toward people with AD. The sample in the current study was representative in terms of the demographic characteristics of the student population. This was evident because the students' demographics were very similar to other studies that were conducted on the same target population in recent years [31–33]. Results from the current study indicated a gap in knowledge among nursing students toward people with AD Conversely, nursing students showed positive attitudes toward people with AD

In the current study, the mean score of the ADKS representing the dementia knowledge level among students was 18.3 out of 30. This score suggests that Jordanian nursing students have a gap in their knowledge regarding AD Similarly, the sub-domains of ADKS also had low scores, ranging from 2.9 out of 4 for the “course of disease” sub-domain to 2.3 out of 5 for the “caregiving” sub-domain. Some studies in the literature reported a higher knowledge mean score, whilst others reported a lower mean score, and all used the same tool. In a study conducted in Malta, nursing students' mean ADKS score was 19.36 out of 30 [34]. Conversely, an Indian study reported a lower mean knowledge score of 16.8 out of 30 [15]. The average American college student’s score was 20.19 out of 30 [28]. It can be noticed that, even though the reported scores were either higher or lower than the current study score, the difference is considerably small and does not exceed 2 points. Such similar results may indicate that poor knowledge among nursing students is a prevalent problem worldwide.

These studies concluded that there is a gap in the students' knowledge regarding AD that must be corrected. This could be related to the lack of educational materials presented to students during their university studies [35]. This conclusion also applies to the current research: only 12% of the students who participated in the study were enrolled in a course that covered subjects related to dementia, and 45% of them had not read any research about dementia at all – keeping in mind that 42.5% of them were fourth-year students, and by this time they should have covered materials regarding AD Also, the ANOVA test did not reveal any significant difference between different academic levels regarding AD knowledge.

The mean score of the DAS in the current study was 91 out of 140. This score skews to the positive side, indicating a positive attitude of nursing students toward people with AD This finding is in line with the literature. Positive attitudes in general toward people with AD were reported in the literature [36]. The positive attitude among Jordanian students could be translated as them having the willingness and potential to care for people living with A.D., but, unfortunately, they may not have adequate knowledge to do so, as seen previously in the results.

Furthermore, there is a consensus in a number of previous studies that a positive attitude was correlated with more experience and clinical exposure to people with dementia [9]. The findings of this current study contradict that consensus. In the current study, the fourth-year students were believed to have more clinical exposure as they have taken more clinical courses and training and thus hold a more positive attitude. However, there was no significant difference between different academic levels according to ANOVA results.

Examining the current study results suggests a gap in knowledge among Jordanian nursing students regarding A.D., but, at the same time, the students had positive attitudes. Their inadequate knowledge could have resulted from insufficient educational material about AD or dementia being offered to them as part of their course curriculum. Positive attitudes in the current study could be attributed to the Jordanian population's respect toward older people as a part of the Jordanian culture [37]. The positive attitude was not attributed to knowledge in this study, as shown as a poor correlation between ADKS scores and DAS scores.

Promoting Excellence framework, which is the theoretical construct used in the current study could be used as a guide to fill this knowledge gap [25]. The framework identifies areas in which dementia knowledge is insufficient through identifying questions with the least correct answer. On the personal level (students) this will guide them to areas in which improvement of knowledge is needed. On the organizational level, nursing curricula could focus on these gaps identified through the survey questions by providing theoretical and clinical courses specifically related to dementia. Further, it is reported in the literature that specialized programs for AD education that include lectures and clinical training could be offered to enhance the students' knowledge [38]. The effect of these programs could be evaluated through research studies or licensure exams. This improvement should not take place just for the sake of improving students' knowledge level, but also to build confidence and competency in dementia care and enhance students' and caregivers' attitudes towards people living with AD disease.

In Jordan, where this study was conducted, student nurses are not widely exposed to people with AD due to the country’s considerably young population compared to other countries with older populations [11, 39]. However, the number of older people is expected to increase in Jordan in the next few years [11]; this will be accompanied by an increase in the number of Alzheimer's cases. Therefore, teachers can use the results from this study to tailor educational interventions to increase the knowledge level among student nurses, thus enhancing their skills and care when dealing with such cases in their future careers. Literature suggests that providing learning opportunities in the field of dementia can equip nursing students with knowledge, positive attitudes, dementia confidence, and competence [9].

### Limitations

One of the most important limitations of this study is the low participation rate. The current study utilized an online questionnaire to collect data about student nurses' knowledge and attitudes. Online methods of collecting data have the inherent limitation of a low response rate. Yet, participants in the online questionnaire will complete the survey based on their willingness to do so, not to please their teachers. This gives more reliable data that reflect the real-life situation. Another limitation was that students were not asked if they had a family member with dementia or if they cared for someone with dementia. Positive responses to either question might affect knowledge or attitude outcomes.

## Conclusion

Nursing students in the current study had insufficient knowledge regarding AD. This may suggest that they are not being provided with educational materials about AD during their undergraduate studies. When they graduate and qualify as registered nurses, this deficient knowledge may be reflected as low quality of care provided to people with AD Conversely, students expressed positive attitudes toward people with AD. This could serve as a platform for these students to absorb information about AD and thus enhance the care of people with AD in their prospective careers. Nursing educators have an excellent opportunity to equip their students with AD knowledge to fill this gap and enhance students' competencies.

## Data Availability

The datasets generated and/or analyzed during the current study are available from the Principal Investigator (Ma’en Aljezawi) on reasonable request.

## Abbreviations

AD: Alzheimer's disease
ADKS: Alzheimer’s Disease Knowledge Scale
DAS: Dementia Attitudes Scale
